# Perillaldehyde Inhibits AHR Signaling and Activates NRF2 Antioxidant Pathway in Human Keratinocytes

**DOI:** 10.1155/2018/9524657

**Published:** 2018-02-14

**Authors:** Yoko Fuyuno, Hiroshi Uchi, Mao Yasumatsu, Saori Morino-Koga, Yuka Tanaka, Chikage Mitoma, Masutaka Furue

**Affiliations:** ^1^Department of Dermatology, Graduate School of Medical Sciences, Kyushu University, 3-1-1 Maidashi, Higashiku, Fukuoka 812-8582, Japan; ^2^Research and Clinical Center for Yusho and Dioxin, Kyushu University Hospital, 3-1-1 Maidashi, Higashiku, Fukuoka 812-8582, Japan; ^3^Division of Statistics, Center for Cohort Studies, Graduate School of Medical Sciences, Kyushu University, 3-1-1 Maidashi, Higashiku, Fukuoka 812-8582, Japan

## Abstract

The skin covers the outer surface of the body, so the epidermal keratinocytes within it are susceptible to reactive oxygen species (ROS) generated by environmental pollutants such as benzo(a)pyrene (BaP), a potent activator of aryl hydrocarbon receptor (AHR). Antioxidant activity is generally mediated by the nuclear factor-erythroid 2-related factor-2 (NRF2) and heme oxygenase-1 (HO1) axis in human keratinocytes. Perillaldehyde is the main component of *Perilla frutescens*, which is a medicinal antioxidant herb traditionally consumed in East Asia. However, the effect of perillaldehyde on the AHR/ROS and/or NRF2/HO1 pathways remains unknown. In human keratinocytes, we found that perillaldehyde (1) inhibited BaP-induced AHR activation and ROS production, (2) inhibited BaP/AHR-mediated release of the CCL2 chemokine, and (3) activated the NRF2/HO1 antioxidant pathway. Perillaldehyde is thus potentially useful for managing inflammatory skin diseases or disorders related to oxidative stress.

## 1. Introduction

*Perilla frutescens* (*shiso* in Japanese) is a medicinal herb traditionally consumed in East Asia. It has recently attracted increasing attention because its major aromatic constituent, perillaldehyde (PAH), exhibits potent antimicrobial [1, 2], antilipidemic [3, 4], anti-inflammatory [5, 6], antioxidant [3], and anticancer activities [7].

The skin covers the outer surface of the body, so the epidermal keratinocytes within it are susceptible to the oxidative stress induced by environmental pollutants such as benzo(a)pyrene (BaP) and dioxins, which cause skin cancer and accelerate skin aging [8, 9]. Oxidative stress is also related to many dermatological diseases including vitiligo and atopic dermatitis [10, 11]. Most free radicals in the body exist in the form of reactive oxygen species (ROS). Excessive free radicals damage not only DNA but also cellular proteins and lipids [12, 13].

It is known that the generation of ROS by BaP and dioxin is mediated, at least in part, by activation of aryl hydrocarbon receptor (AHR) [8, 14]. AHR is a xenobiotic chemical sensor abundantly expressed in the epidermal keratinocytes [15, 16]. Upon ligation, the activated AHR translocates from the cytoplasm into the nucleus. This translocated AHR binds to its specific DNA recognition site, namely, xenobiotic-responsive element, and upregulates the transcription of responsive genes, such as cytochrome P450 1A1 (CYP1A1) [15–17]. The enhanced production of CYP1A1 metabolizing enzyme leads to the generation of ROS and may damage proteins and DNA [18–20].

The excessive production of ROS should be neutralized or minimized by antioxidants in order to maintain skin homeostasis. Antioxidant enzymes such as heme oxygenase-1 (HO1) play a pivotal role in ameliorating oxidative stress in keratinocytes [9, 21]. The induction of these antioxidant enzymes is regulated by nuclear factor-erythroid 2-related factor-2 (NRF2), which is a master switch for antioxidant signaling [9, 22, 23]. Under physiological conditions, the level of NRF2 in the cytoplasm is regulated by the formation of the NRF2-KEAP1-CUL3 complex [23]. Under oxidative conditions, NRF2 dissociates from KEAP1 and the free NRF2 translocates to the nucleus and initiates transcription of the antioxidant gene HO1 [23].

To protect the skin against ROS formation, natural edible antioxidants are particularly interesting because of a safety perspective [21]. However, the molecular mechanism behind the antioxidative effects of PAH remains unknown. To bridge this research gap, in this study, we demonstrate that PAH inhibits AHR/CYP1A1/ROS signaling and upregulates the NRF2/HO1 antioxidant pathway in human keratinocytes.

## 2. Materials and Methods

### 2.1. Reagents and Antibodies

The sources from which reagents and antibodies were purchased for this study are listed in Table 1. PAH stock solution was dissolved in ethanol at a final concentration of up to 1000 mM. BaP stock solution was dissolved in dimethyl sulfoxide (DMSO) at a final concentration of 1 mM. Various concentrations of PAH (up to 1000 *μ*M) and BaP (1 *μ*M) were prepared in cell culture medium. Control cultures received medium containing comparable amounts of DMSO (0.1%) and ethanol (0.1%).

### 2.2. Cell Culture

HaCaT cells, a human keratinocyte cell line, were maintained in Dulbecco's modified Eagle's medium containing 10% fetal bovine serum and antibiotics in culture dishes at 37°C in 5% CO_2_. Culture medium was replaced every 2-3 days. Upon approaching confluence (70%–90%), cells were washed two times with phosphate-buffered saline (PBS), disaggregated with 0.05% (*w*/*v*) trypsin-0.53 mM EDTA-4Na solution and subcultured. HaCaT cells at the 16th to 21st passages were used in all experiments. HaCaT cells (2.0 × 10^5^) were seeded in six-well culture plates and allowed to attach for 48 h, for real-time quantitative RT-PCR, siRNA transfection, and ELISA. HaCaT cells (3.0 × 10^4^) were also seeded in an eight-well slide for 24 h for immunofluorescence. NRF2 siRNA and control siRNA were transfected into HaCaT cells using Lipofectamine® RNAiMAX Reagent (RNAiMAX), in accordance with the manufacturer's instructions. HaCaT cells cultured in six-well plates were incubated with RNAiMaX containing 25 pmol siRNA and 2.5 *μ*l RNAiMAX in 1 ml of culture medium. After a 48 h incubation period, siRNA-transfected HaCaT cells were treated with PAH for 5 h.

### 2.3. Real-Time Quantitative RT-PCR

We followed the methods of Uchi et al. HaCaT cells were treated with BaP (1 *μ*M) in the presence or absence of PAH (100 *μ*M) for 5 h [8, 22]. BaP and PAH were added to the cell culture at the same time. Total RNA was then isolated from cells using the RNeasy® Mini kit. Quantitative real-time RT-PCR was performed with PrimeScript™ RT reagent and SYBR® Premix Ex Taq™, in accordance with the manufacturer's instructions. PCR amplification was performed with the following cycling conditions: 95°C for 30 s, then 40 cycles of 95°C for 5 s (denaturation), and 60°C for 20 s (annealing/extension). The cycle threshold (Ct) for each amplification was normalized to that of *ACTB* (internal control). Normalized gene expression is expressed as the quantity of gene-specific mRNA in each treatment group relative to that in the control group (fold induction). Oligonucleotide primers were as follows:

*CYP1A1*: forward 5′-TAGACACTGATCTGGCTGCAG-3′ and reverse 5′-GGGAAGGCTCCATCAGCATC-3′; *CCL2*: forward 5′-CCCCAGTCACCTGCTGTTAT-3′ and reverse 5′-TGGAATCCTGAACCCACTTC-3′; *HO1*: forward 5′-AAGACTGCGTTCCTGCTCAAC-3′ and reverse 5′-AAAGCCCTACAGCAACTGTCG-3′; *NRF2*: forward 5′-TCAGCGACGGAAAGAGTATGA-3′ and reverse 5′-CCACTGGTTTCTGACTGGATGT-3′; *IL1B*: forward 5′-ATGATGGCTTATTACAGTGGCAA-3′ and reverse 5′-GTCGGAGATTCGTAGCTGGA-3′; and *ACTB*: forward 5′-ATTGCCGACAGGATGCAGA-3′ and reverse 5′-GAGTACTTGCGCTCAGGAGGA-3′.

### 2.4. Immunofluorescence

We followed the methods of Uchi et al. HaCaT cells were plated on an eight-well slide and were treated with or without PAH (100 *μ*M) and/or BaP at subconfluence for 1 h (for NRF2) or 5 h (for AHR). Then, cells were washed with PBS, fixed with acetone for 10 min, and blocked with 5% bovine serum albumin in PBS for 30 min. Samples were incubated with primary rabbit anti-AHR antibody or rabbit anti-NRF2 antibody. Specific binding was detected using horseradish peroxidase-conjugated green-fluorescent Alexa Fluor® 488, in accordance with the manufacturer's protocol. Samples were covered with UltraCruz™ mounting medium containing 4′,6-diamidino-2-phenylindole. The proportion of cells showing nuclear-dominant staining for AHR or NRF2 was calculated in three different high-power fields and averaged.

### 2.5. Detection of ROS Production

We followed the methods of Uchi et al. HaCaT cells were plated on an eight-well slide and were treated with PAH (100 *μ*M) with or without BaP at subconfluence for 24 h. An oxidation-sensitive dye, carboxy-H2DCFDA, was used to quantify ROS levels in live cells. Cells were incubated with Hank's balanced saline solution (HBSS) containing carboxy-H_2_DCFDA (25 *μ*M) for 30 min at 37°C. The fluorescence images were acquired using an EVOS^R^ FL cell imaging system (Life Technologies, Carlsbad, CA, USA). The relative fluorescence intensity was quantified using ImageJ (National Institutes of Health, Rockville, MD, USA) [24].

### 2.6. ELISA

Cell culture supernatants were cleared by centrifugation and analyzed for the presence of immunoreactive CCL2 protein using the Quantikine Human CCL2/MCP1 ELISA Kit and IL-1*β* protein using the Quantikine Human IL-1*β* ELISA Kit, in accordance with the manufacturer's instructions. Absorbance was measured using an iMark microplate absorbance reader (Bio-Rad, Hercules, CA, USA), and the concentrations of the chemokines were determined in each sample by comparison to a standard curve.

### 2.7. Measurement of ROS by Flow Cytometry

The formation of ROS was measured on a BD FACS Canto™ II flow cytometer (BD Biosciences, Franklin Lakes, NJ, USA). Briefly, HaCaT cells (1.2 × 10^5^ cells/ml) were suspended in 1 ml of medium and incubated with PAH (100 *μ*M) in the presence or absence of BaP (1 *μ*M) for 24 h at 37°C. They were washed twice with HBSS and incubated with carboxy-H2DCFDA at a final concentration of 25 *μ*M for an additional 30 min at 37°C in the dark. Intracellular ROS resulted in an increase in fluorescence as measured by flow cytometry.

### 2.8. Statistical Analysis

Data are presented as mean ± standard error (S.E.). The significance of differences between groups was assessed using Student's unpaired two-tailed *t*-test (when two groups were analyzed) or one-way analysis of variance (for three or more groups). A *p* value of less than 0.05 was considered statistically significant.

## 3. Results

### 3.1. Perillaldehyde Did Not Affect the Viability of Keratinocytes

Cell viability was determined by the CCK-8 assay. PAH (up to 1000 *μ*M) alone or in the presence of BaP did not affect the viability of HaCaT cells (Supplementary Figures S1A and S1B).

### 3.2. Perillaldehyde Diminished *CYP1A1* Expression Induced by BaP

**Figure 1:**
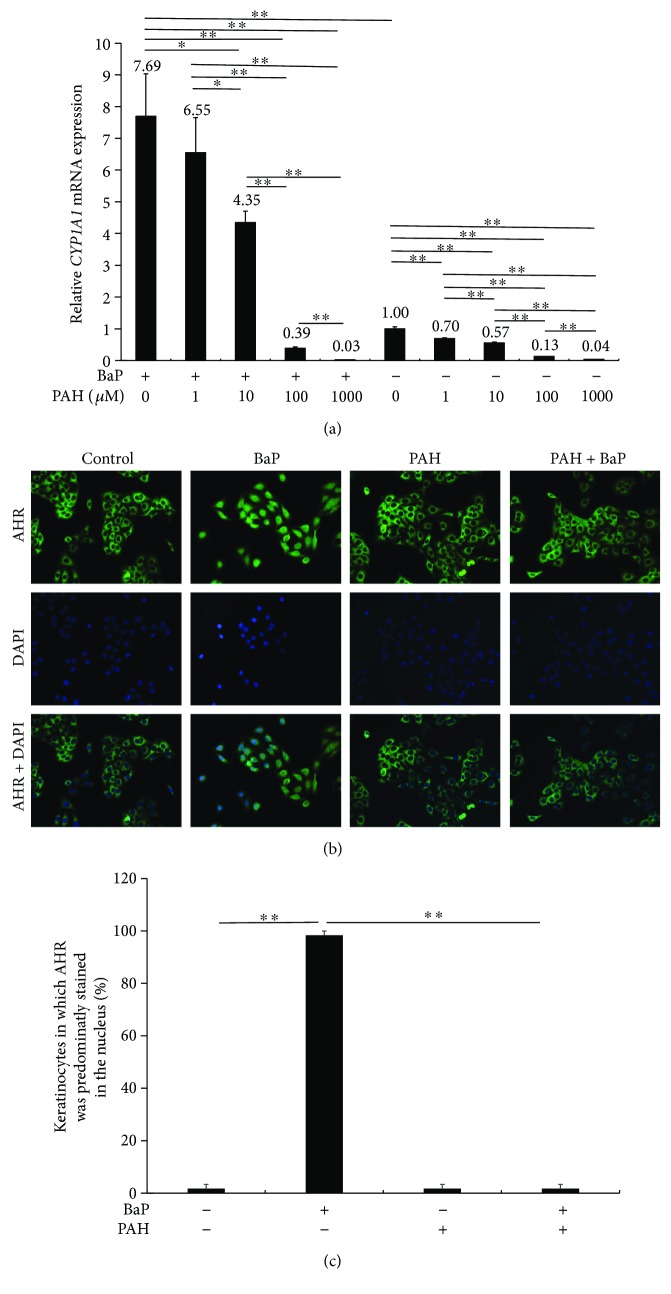
(a) HaCaT keratinocytes were treated with BaP in the presence or absence of PAH (1, 10, 100, or 1000 *μ*M), and *CYP1A1* expression was measured by quantitative RT-PCR. ^∗^ *p* < 0.05. ^∗∗^ *p* < 0.01. (b) Subcellular localization of AHR was visualized by immunofluorescence. Cells were stained with anti-AHR antibody (green) and 4′,6-diamidino-2-phenylindole (blue). BaP induced cytoplasmic to nuclear translocation of AHR, which was blocked by PAH. (c) The proportion of keratinocytes in which AHR was predominantly stained in the nucleus was calculated. ^∗∗^ *p* < 0.01.

HaCaT cells were treated with 1 *μ*M BaP in the presence or absence of PAH (0, 1, 10, 100, and 1000 *μ*M), and then total RNA was extracted for qRT-PCR analysis. BaP significantly upregulated *CYP1A1* expression, but PAH dose-dependently decreased the induction of *CYP1A1* expression by BaP (Figure 1(a)). Notably, PAH also inhibited the baseline expression of *CYP1A1* in a dose-dependent manner (Figure 1(a)).

Upon activation by BaP, cytoplasmic AHR is known to translocate to the nucleus to induce the transcription of CYP1A1 [8]. Therefore, we investigated whether PAH inhibits BaP-induced AHR nuclear translocation. In untreated control and PAH-treated keratinocytes, AHR was mainly located in the cytoplasm (Figure 1(b)). Upon BaP treatment, AHR translocated into the nucleus; however, PAH inhibited the BaP-induced nuclear translocation of AHR (Figures 1(b) and 1(c)). These results indicate that PAH effectively inhibited AHR signaling.

### 3.3. Perillaldehyde Reduced CCL2 Expression Induced by BaP

**Figure 2:**
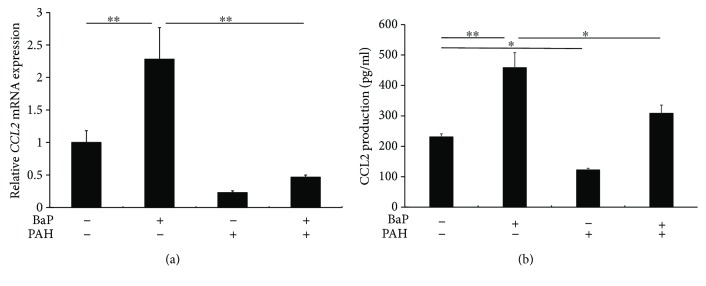
(a) BaP significantly upregulated *CCL2* expression, which was blocked in the presence of PAH. ^∗∗^ *p* < 0.01. (b) BaP significantly increased the production of CCL2 protein, which was also inhibited by PAH. ^∗^ *p* < 0.05. ^∗∗^ *p* < 0.01.

Since BaP stimulates the production of CCL2, which is upregulated in the lesional skin in atopic dermatitis and psoriasis [25–27], we next examined whether PAH inhibits BaP-induced *CCL2* expression. As has been reported previously [25], BaP upregulated the mRNA expression of *CCL2*, which was downregulated by PAH (Figure 2(a)). PAH also inhibited the BaP-induced release of CCL2 protein in keratinocytes (Figure 2(b)).

The mRNA levels of *IL1B* were also upregulated by BaP exposure, and PAH inhibited the BaP-induced *IL1B* expression, while the protein level of IL-1*β* was not altered by stimulation with BaP or PAH (Supplementary Figure S2). We speculated that the overall levels of IL-1*β* were very low and no inflammasome-activating conditions were used that would lead to IL-1*β* secretion.

### 3.4. Perillaldehyde Induced NRF2 Nuclear Translocation and HO1 Expression

**Figure 3:**
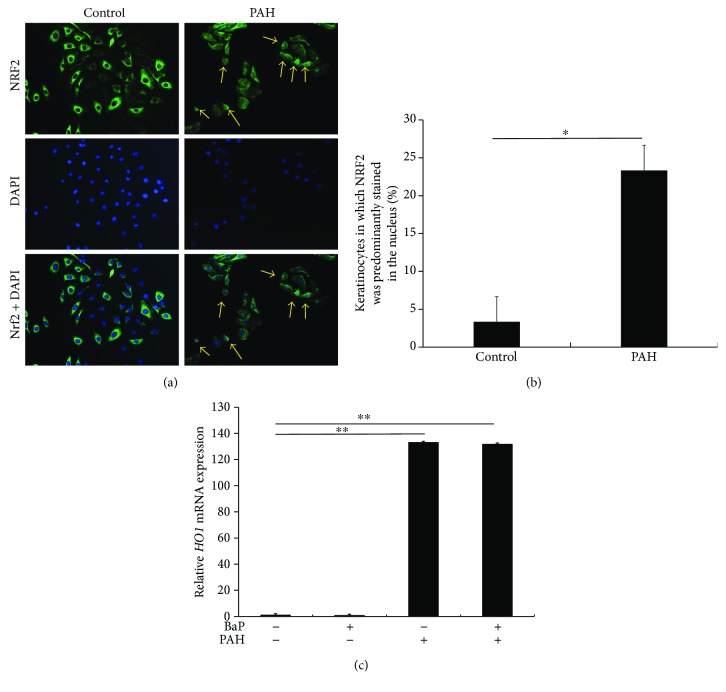
(a) HaCaT keratinocytes were stained with anti-NRF2 antibody (green) and 4′,6-diamidino-2-phenylindole (blue). NRF2 was mainly localized in the cytoplasm in untreated cells. PAH induced cytoplasmic to nuclear translocation of NRF2 (arrows). (b) The proportion of keratinocytes in which NRF2 was predominantly stained in the nucleus was calculated. ^∗^ *p* < 0.05. (c) Transcription of *HO1* mRNA was significantly upregulated by PAH. BaP did not affect *HO1* expression. ^∗∗^ *p* < 0.01.

As PAH exerts antioxidant activity [3], we next examined whether it activates and induces the nuclear translocation of NRF2. In control keratinocytes, NRF2 was mainly localized in the cytoplasm, while it translocated into the nucleus upon stimulation with PAH (Figures 3(a) and 3(b)). In parallel with the nuclear translocation of NRF2, the expression of *HO1* was significantly upregulated by PAH. BaP did not induce *HO1* expression or downregulate PAH-induced *HO1* upregulation (Figure 3(c)).

**Figure 4:**
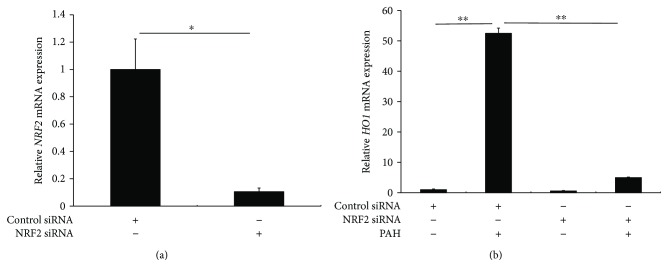
(a) Transfection of NRF2 siRNA significantly reduced *NRF2* expression. ^∗^ *p* < 0.05. (b) PAH-induced *HO1* upregulation was canceled in keratinocytes with NRF2 knockdown. ^∗∗^ *p* < 0.01.

To prove the dependence of PAH-induced *HO1* upregulation on NRF2, NRF2 was knocked down by transfection with NRF2 siRNA (Figure 4(a)). PAH-mediated *HO1* upregulation was canceled in keratinocytes with NRF2 knockdown (Figure 4(b)).

### 3.5. Perillaldehyde Suppressed BaP-Induced ROS Expression

**Figure 5:**
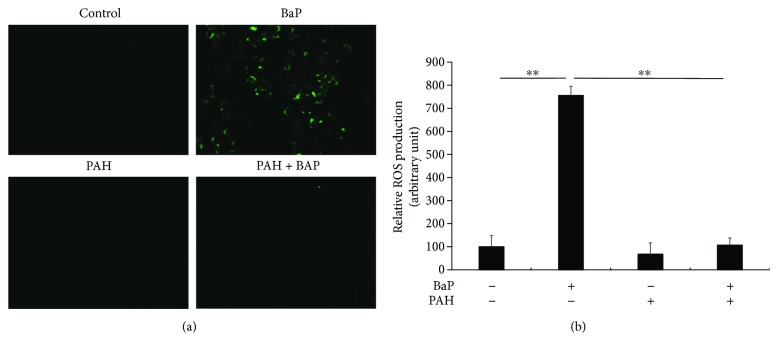
(a) ROS production was visualized by a fluorescence technique using carboxy-H_2_DCFDA. BaP induced ROS production in keratinocytes. However, this was inhibited by the simultaneous presence of PAH. (b) Relative ROS production was quantified by fluorescence intensity. ^∗∗^ *p* < 0.01.

Since PAH upregulated the antioxidant NRF2/HO1 pathway, we next investigated whether PAH is capable of inhibiting the ROS production induced by BaP exposure. As has been reported previously [8, 22], BaP induced ROS production in the keratinocytes (Figure 5(a)). PAH itself did not induce ROS production, while the presence of PAH in combination with BaP potently inhibited the ROS production induced by the latter (Figures 5(a) and 5(b), Supplementary Figure S3).

## 4. Discussion

Smoking is a significant risk factor for developing inflammatory skin diseases such as atopic dermatitis and psoriasis [28, 29]. BaP is a major pollutant present in tobacco smoke [8]. The present and previous studies showed that BaP activated AHR signaling and induced the production of ROS and the release of CCL2 [8, 25]. Oxidative stress and subsequent production of inflammatory chemokines are thought to be critical mediators in the initiation and exacerbation of inflammatory skin diseases [9, 11, 25–27, 30, 31].

Several classes of beneficial dietary phytochemicals, such as polyphenols and glucosinolates, have been described as health-promoting or disease-preventing. Interestingly, many dietary compounds from vegetables and fruit with anti-inflammatory properties have been found to be AHR ligands or NRF2 activators [21, 32]. *Perilla frutescens* and its major constituent PAH have also been reported to possess hypolipidemic, anti-inflammatory, neuroprotective, antidepressant-like, and antifungal effects [1–7]. There are currently only limited pharmacokinetic data on PAH formulations, especially the tissue distribution. Omari-Siaw et al. reported pharmacokinetic parameters of PAH in mouse tissues after the oral administration of 240 mg/kg PAH. AUC_0–4_ in the plasma was 3152.48 ± 35.36 ngh/ml, in the liver 230.24 ± 54.13 ngh/ml, in the lungs 246.49 ± 19.53 ngh/ml, in the spleen 251.04 ± 41.03 ngh/ml, in the kidney 193.34 ± 87.98 ngh/ml, in the brain 194.40 ± 48.91 ngh/ml, and in the heart 167.82 ± 12.11 ngh/ml. PAH accumulates at a high level in the liver [3].

In the present study, PAH inhibited BaP-induced *CYP1A1* upregulation in a dose-dependent fashion in keratinocytes. In addition, PAH itself reduced the baseline expression of *CYP1A1*. Since the regulation of *CYP1A1* expression is a specific event for AHR activation [8, 15], PAH is considered a potent inhibitor of both baseline and inducible AHR activations. In accordance with this, PAH appeared to inhibit the BaP-induced nuclear translocation of AHR. PAH also inhibited the BaP-induced CCL2 transcription and protein upregulation. CCL2 plays a crucial role in inflammatory diseases [33], and it is known that another pollutant dioxin increases CCL2 production via AHR activation [33, 34]. The potent inhibitory action of PAH on AHR activation may be beneficial for preventing inflammation mediated by pollutants and AHR.

We next elucidated that PAH activated the NRF2/HO1 antioxidant pathway. PAH induced NRF2 nuclear translocation and subsequent *HO1* upregulation. Moreover, PAH potently inhibited BaP-mediated ROS production in keratinocytes. NRF2 orchestrates antioxidant activity as a nuclear transcription factor by upregulating a series of antioxidant enzymes [35, 36]. The present study also demonstrated that PAH-induced *HO1* upregulation was dependent on NRF2 activation because it was canceled in keratinocytes with NRF2 knockdown. Environmental pollutants induce oxidative stress and accelerate skin aging, leading to skin inflammation and carcinogenesis [9, 15]. Similar to other antioxidant phytochemicals [14, 21, 37–39], PAH exerts its antioxidant activity via NRF2/HO1 signaling and dampens ROS production mediated by pollutants and AHR.

## 5. Conclusions

In conclusion, PAH may protect keratinocytes from BaP-AHR-mediated oxidative stress and CCL2 production by downregulating AHR signaling and upregulating the NRF2/HO1 antioxidant pathway. Although further studies are needed, the dual effects of PAH may be valuable in protecting against the inflammatory process induced by pollutants and AHR.

## Figures and Tables

**(a):** 

Reagent	
Perillaldehyde (PAH)	Tokyo Chemical Industry (Tokyo, Japan)
Dulbecco's modified Eagle's medium	Sigma-Aldrich (St. Louis, MO, USA)
Benzo(a)pyrene (BaP)	
Dimethyl sulfoxide (DMSO)	
Ethanol	Wako Laboratory Chemicals (Osaka, Japan)
0.05% (*w*/*v*) trypsin-0.53 mM EDTA-4Na solution	

**(b):** 

Antibody	
Anti-AHR rabbit polyclonal antibody (H-211)	Santa Cruz Biotechnology (Dallas, TX, USA)
Anti-NRF2 rabbit polyclonal antibody (H-300)	
UtraCruzTM mounting medium containing 4′,6-diamidino-2-phenylindole	
Horseradish peroxidase-conjugated green-fluorescent Alexa Flour® 488	Molecular Probes (Eugene, OR, USA)
5-(and −6)-Carboxy-2′,7′-dichlorodihydrofluorescein diacetate (carboxy-H2DCFDA)	

**(c):** 

Kit	
RNeasy Mini kit	Qiagen (Hilden, Germany)
PrimeScriptTM RT reagent	Takara Bio (Kusatsu, Japan)
SYBR® Premix Ex TaqTM II	
Quantikine human CCL2/MCP1 ELISA kit	R&D Systems (Minneapolis, MN, USA)
Quantiline human IL-1*β* ELISA kit	
CCK-8 assay kit	Dojindo Laboratories (Kumamoto, Japan)

**(d):** 

siRNA	
NRF2 siRNA (s9492)	Ambion (Austin, TX, USA)
Control siRNA (negative control number 1)	
Lipofectamine® RNAiMAX Reagent (RNAiMAX)	Invitrogen™ (Carlsbad, CA, USA)

## Supplementary Materials

Supplementary 1 Figure S1: (A) PAH (up to 1000 *μ*M) alone did not affect the viability of HaCaT cells. (B) PAH (up to 1000 *μ*M) in the presence of BaP (1 *μ*M) did not affect the viability of HaCaT cells.

Supplementary 2 Figure S2: (A) BaP upregulates the *IL1B* expression and it is inhibited in the presence of PAH in HaCaT keratinocytes. ^∗∗^ *p* < 0.01. (B) However, the production of IL-1*β* protein is not affected by BaP and PAH.

Supplementary 3 Figure S3: flow cytometric analysis of ROS confirmed the inhibitory action of PAH against BaP-induced ROS generation. ^∗∗^ *p* < 0.01.
