# Corrigendum to “Perillaldehyde Inhibits AHR Signaling and Activates NRF2 Antioxidant Pathway in Human Keratinocytes”

**DOI:** 10.1155/2018/6091947

**Published:** 2018-05-22

**Authors:** Yoko Fuyuno, Hiroshi Uchi, Mao Yasumatsu, Saori Morino-Koga, Yuka Tanaka, Chikage Mitoma, Masutaka Furue

**Affiliations:** ^1^Department of Dermatology, Graduate School of Medical Sciences, Kyushu University, 3-1-1 Maidashi, Higashiku, Fukuoka 812-8582, Japan; ^2^Research and Clinical Center for Yusho and Dioxin, Kyushu University Hospital, 3-1-1 Maidashi, Higashiku, Fukuoka 812-8582, Japan; ^3^Division of Statistics, Center for Cohort Studies, Graduate School of Medical Sciences, Kyushu University, 3-1-1 Maidashi, Higashiku, Fukuoka 812-8582, Japan

In the article titled “Perillaldehyde Inhibits AHR Signaling and Activates NRF2 Antioxidant Pathway in Human Keratinocytes” [1], there was an error in Supplementary Figure S3. The figure incorrectly showed that PAH induced ROS expression and BaP suppressed ROS expression induced by PAH, whereas the corrected figure shows that BaP induced ROS expression and PAH suppressed ROS expression induced by BaP. The correct supplementary figure is available here.

## Supplementary Materials

Supplementary Materials Supplementary 3. Figure S3: flow cytometric analysis of ROS confirmed the inhibitory action of PAH against BaP-induced ROS generation. ^∗∗^ *p* < 0 01.
