# Supplementary figures and images for: Corrigendum to “Perillaldehyde Inhibits AHR Signaling and Activates NRF2 Antioxidant Pathway in Human Keratinocytes”
Source: Oxid Med Cell Longev. 2018 May 22;2018:6091947. doi: 10.1155/2018/6091947 (PMC5987232; doi:10.1155/2018/6091947)

S3

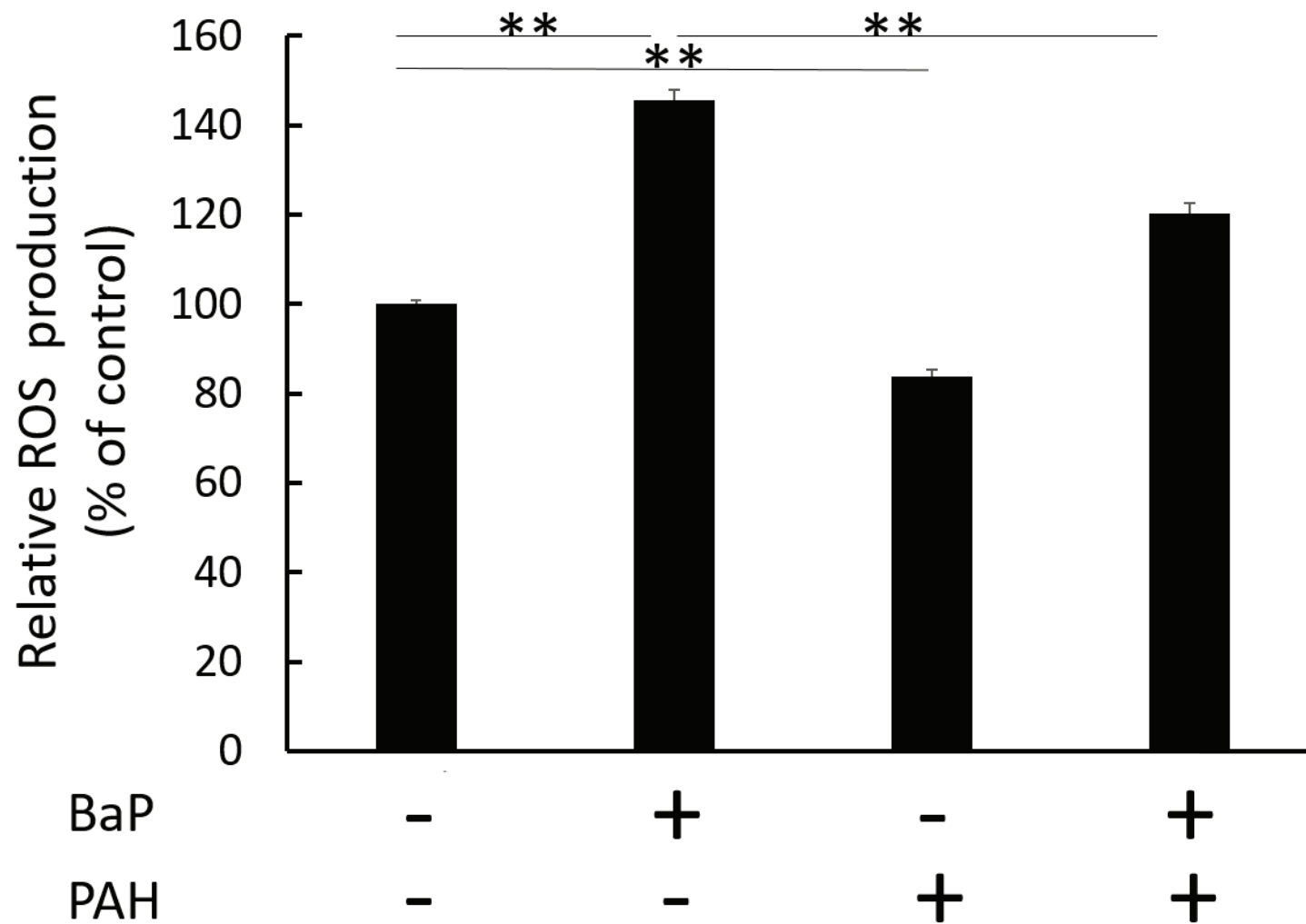

Supplement: Supplementary Materials — Supplementary 3. Figure S3: flow cytometric analysis of ROS confirmed the inhibitory action of PAH against BaP-induced ROS generation. ∗∗ p < 0 01. [file 6091947.f1.pdf]
